# Molecular dynamics modeling of the *Vibrio cholera* Na^+^-translocating NADH:quinone oxidoreductase NqrB–NqrD subunit interface

**DOI:** 10.1007/s11010-021-04266-3

**Published:** 2021-10-09

**Authors:** Alexander Dibrov, Muntahi Mourin, Pavel Dibrov, Grant N. Pierce

**Affiliations:** 1 Department of Family Medicine, Max Rady College of Medicine, University of Manitoba, Winnipeg, MB Canada; 2 Department of Physiology and Pathophysiology, Faculty of Health Sciences, University of Manitoba, Winnipeg, MB Canada; 3 Department of Microbiology, Faculty of Sciences, University of Manitoba, Winnipeg, MB Canada; 4 Albrechtsen Research Centre, St. Boniface Hospital, 351 Taché Avenue, Winnipeg, MB Canada

**Keywords:** Na^+^-NQR, *Vibrio cholerae*, Structure, Molecular dynamics, GROMACS, FREAD

## Abstract

The Na^+^-translocating NADH:quinone oxidoreductase (Na^+^-NQR) is the major Na^+^ pump in aerobic pathogens such as *Vibrio cholerae*. The interface between two of the NQR subunits, NqrB and NqrD, has been proposed to harbor a binding site for inhibitors of Na^+^-NQR. While the mechanisms underlying Na^+^-NQR function and inhibition remain underinvestigated, their clarification would facilitate the design of compounds suitable for clinical use against pathogens containing Na^+^-NQR. An in silico model of the NqrB–D interface suitable for use in molecular dynamics simulations was successfully constructed. A combination of algorithmic and manual methods was used to reconstruct portions of the two subunits unresolved in the published crystal structure and validate the resulting structure. Hardware and software optimizations that improved the efficiency of the simulation were considered and tested. The geometry of the reconstructed complex compared favorably to the published *V. cholerae* Na^+^-NQR crystal structure. Results from one 1 µs, three 150 ns and two 50 ns molecular dynamics simulations illustrated the stability of the system and defined the limitations of this model. When placed in a lipid bilayer under periodic boundary conditions, the reconstructed complex was completely stable for at least 1 µs. However, the NqrB–D interface underwent a non-physiological transition after 350 ns.

## Introduction

The Na^+^-translocating NADH:quinone oxidoreductase (Na^+^-NQR) is the major Na^+^ pump in aerobic pathogens such as the human pathogen *Vibrio cholerae*. Na^+^-NQR oxidizes NADH, feeding electrons into the respiratory chain at the quinone level. The enzyme transports Na^+^ from the cytoplasm, thus generating bacterial energy through a sodium motive force (SMF) [1, 2]. Na^+^-NQR is widely distributed amongst different pathogenic species. Besides *V. cholerae*, the Na^+^-NQR is present in *Neisseria gonorrhoeae*, *Klebsiella pneumoniae*, *Pseudomonas aeruginosa*, *Porphyromonas gingivalis* and many other pathogens [2–4]. The SMF generated by Na^+^-NQR is necessary for the pathogenicity, motility, virulence and antibiotic resistance of pathogenic bacteria [1–3]. The exploitation of sodium electrochemical gradient for energy generation is advantageous for pathogens residing in the blood plasma or gut, as both environments are enriched in sodium. The presence of bile salts and fatty acids in the gut, as well as high pH within the lumen of the distal ileum and the mucosa of the human colon [2, 5], make these environments particularly hostile to respiratory processes that utilize transmembrane proton gradient. Despite this, the Na^+^-NQR is not widely represented in the commensal gut microbiota. This makes the Na^+^-NQR an attractive target for antimicrobial agents that can be less damaging to human intestinal flora [6]. Most importantly, the Na^+^-NQR is absent in the eukaryotic cells. This makes the Na^+^-NQR especially attractive as a target for prospective antibiotics development [2].

The first X-ray diffraction-based crystal structure of the entire Na^+^-NQR complex from *V. cholerae* was reported at 3.5 Å by Steuber et al*.* in 2014 [7]. The availability of such a structure has opened opportunities for the systematic design of inhibitors against Na^+^-NQR. This crystal structure revealed a *V. cholerae* Na^+^-NQR comprised of six subunits, termed NqrA through F. NqrB, NqrD and NqrE are highly hydrophobic subunits embedded in the membrane core. NqrC (located at the periplasmic side), NqrA and NqrF (located at cytoplasmic side) are the three hydrophilic subunits.

According to widely accepted views [7, 8], NADH oxidation occurs at the [2Fe–2S] redox center located within NqrF. Subsequent oxidation of NqrF creates conformational changes that transfer the electron to the [Fe–S] center at the NqrD–E interface. This event facilitates the binding of Na^+^ to the NqrB subunit. The negatively charged side chain of Asp346 in the NqrB subunit coordinates Na^+^ upon its entry. The electron is then transferred from a flavin mononucleotide (FMN) associated with NqrC to the FMN of NqrB, presumably triggering the occlusion of Na^+^ within the NqrB subunit. The electron is then transferred from the FMN to the riboflavin of NqrB. This redox step is thought to directly facilitate Na^+^ translocation into the periplasmic space. Local change in the electrostatic environment within the subunit precipitates a conformational change, allowing Na^+^ to escape into the wide periplasmic half-channel. The subsequent electron transfer from riboflavin to ubiquinone (UQ) in NqrB resets the catalytic cycle within this subunit. While this final step is not essential for Na^+^ translocation, it ultimately allows for the generation of a transmembrane sodium gradient [7, 8]. In the NqrB subunit, glycine 140 and glycine 141 are functionally implicated in the binding of ubiquinone [8]. Negatively charged residues D133 in NqrD, D346, E28, E144, Q88 in NqrB and E95 in NqrE are suggested to form a putative Na^+^ binding site [8–10].

Korormicin, a naturally occurring antibiotic produced by the *Pseudomonas* species, is a quinone analog that prevents the Na^+^-NQR dependent formation of SMF [11, 12]. It binds to the NqrB subunit in a non-competitive manner and inhibits the reduction of ubiquinone, possibly by inducing a conformational change [13, 14]. Since the binding of korormicin to NQR interferes with the natural electron transfer, it is provokes a leakage of electrons from reduced redox centers of the enzyme to oxygen and ultimately results in the production of reactive oxygen species (ROS) [15]. The Gly140 is important for the binding of korormicin to NqrB, as a single substitution at the position of Gly140 in NQR causes resistance to korormicin [13]. In 2017, Tuz et al. [16] identified a unique site at the subunit B and D interface important for ubiquinone binding. Mutations in P185, L190 and F193 residues in subunit D open the interface between the B and D subunits which allows inhibitor binding and hence inhibition of NQR. Although docking studies by Raba et al. [17] examined possibilities for inhibitor binding at the NqrB–D interface, the exact mechanism of action of korormicin against NQR has not been identified.

The epoxy group in korormicin is a potent source of carcinogenicity, precluding its clinical use in humans [2, 18]. Although korormicin cannot be used due to this toxicity, understanding the mechanism of NQR inhibition by korormicin will facilitate the design of NQR inhibitors with less mammalian toxicity and higher affinity to the bacterial target. Such novel antimicrobials represent important candidates to narrowly target NQR-containing pathogens [2]. For example, recently, we have used korormicin as a parent compound to design an NQR inhibitor, named PEG-2S. The 11-carbon aliphatic tail of korormicin is truncated to seven carbon atoms in PEG-2S, and the epoxy group of the parent compound was eliminated to reduce toxicity. PEG-2S showed greater anti-chlamydial activity and less toxicity than korormicin in vitro [18]. This newly designed compound represents a platform for the development of clinically viable Na^+^-NQR inhibitors. A better understanding of the molecular mechanism underlying the binding of korormicin to Na^+^-NQR would facilitate the further rational design of additional Na^+^-NQR inhibiting compounds.

In order to investigate the mechanism of inhibition by korormicin in silico, the construction of a membrane-bound model of the NqrB and NqrD subunits was achieved. In this study, a combination of algorithmic and manual methods was used to reconstruct missing portions of the two subunits and validate the resulting structure. Special attention was paid to residues thought to be involved in ubiquinone binding at the NqrB–D interface and the stability of the reconstructed complex subsequently demonstrated. The use of a spatially abbreviated unit cell with a simplified membrane composition was also validated. In addition, GPU acceleration with halo exchange was successfully employed for the simulation of this molecular dynamics system. The limitations of our model and potential future refinements are discussed.

## Methods

### Software and hardware for molecular dynamics

Molecular dynamics simulations were performed using a mixed-precision, GPU-accelerated GROMACS [19, 20] 2020.3 build [21]. The software was compiled and run on a Titan S275 scientific research server with two AMD EPYC Rome 7282 2.8 GHz 16-core CPUs and two NVIDIA GeForce RTX 2080ti GPUs. This hardware configuration represents a relative abundance of GPU to CPU computing power, so GPU acceleration was used for all simulations. Optimization passes revealed that it was most efficient to offload as many routines as possible to the GPU, including the computation of bonded interactions. Halo exchange was enabled after an NV Link SLI Bridge was installed to directly connect the GPUs. This allowed for coordinate update tasks to be carried out on GPU in simulations that were parallelized over two GPUs and all available CPU cores.

### Reconstruction of the NqrB–D complex

The crystal structure of the *V. cholerae* NQR complex was obtained from the RCSB Protein Data Bank [22]. These data are missing some side chain information, and backbone coordinates are absent for a number of loop structures. The loops were rebuilt with PyFREAD, a fragment-based loop modeling algorithm implemented in Python that uses a database search method [23]. The May 2017 membrane protein loop database available on the SAbPred website [24] was used in conjunction with the algorithm. The original code was modified to resolve case sensitivity issues related to the database format and improve exception handling. PyFREAD generated multiple decoys representing various completions of a given loop. These were all visually inspected, with a final variant chosen that minimized discontinuities while avoiding steric clashes and perturbation of the known crystal structure. Initial backbone coordinates for three loops on NqrB and four on NqrD were generated in this manner.

Despite careful selection of suitable decoys, the loops as generated using PyFREAD were prone to internal steric clashes and discontinuity with the rest of the protein structure. They also lacked side chain information. To rebuild side chains and address structural errors in loop generation, the coordinates were manually corrected in a virtual reality environment using the Nanome [25] software. Overlapping loop backbone elements were separated in a manner that resolved the overlap without compromising structural integrity. Abnormally long bond distances were resolved by distributing the excess length among nearby residues. As side chains were added, they were rotated to avoid steric clashes and optimize contacts. Short, local steepest descents energy minimizations were performed during this process to ensure that a chemically reasonable structure was maintained. Following a round of energy minimization, the final structure was suitable for all-atom molecular dynamics. Six molecular dynamics simulations were subsequently prepared. Their physical characteristics are summarized in Table 1.

**Table 1:** Physical characteristics of the six molecular dynamics simulations

System	Size		Lipids		Ions
	Atoms	Waters	Composition	Arrangement	
C	150,800	34,730	4:1 POPE:POPG	Bilayer	0.15 M KCl
R1	92,936	19,679	POPC	Bilayer	0.15 M KCl
R2	92,936	19,679	POPC	Bilayer	0.15 M KCl
R3	92,936	19,679	POPC	Bilayer	0.15 M KCl
M1	68,560	14,983	POPC	Arbitrary	Neutralization
M2	114,320	21,107	POPC	Arbitrary	Neutralization

Since the phospholipid composition of the CHARMM system was distinct and box volume varied with barostat coupling, the total number of atoms and total number of water molecules are presented as proxies for system size and composition

### CHARMM system preparation: simulation ‘C’

CHARMM-GUI protocols [26] were used to embed the reconstructed NqrB–D complex in a bilayer membrane composed of mixed 1-palmitoyl-2-oleoylphosphatidylethanolamine (POPE) (80%) and 1-palmitoyl-2-oleoylphosphatidylglycerol (POPG) (20%) [27]. This composition was chosen to mimic the *V. cholerae* cell membrane [26]. The system was solvated with TIP3P water, and potassium chloride was introduced to an approximate 150 mM concentration. The water, lipid and protein components were energy minimized and equilibrated using CHARMM-GUI scripts and settings. These were minimally modified to optimize performance on our hardware and allow for GPU acceleration. A 1.625 ns equilibration procedure was performed at 303.15°K following steepest descents minimization to machine precision. The integration timestep was increased from 1 to 2 fs after the first 375 ps of equilibration, and position restraints were gradually loosened.

### OPLS-AA/L membrane-embedded system preparation: simulations ‘R1–3’

The reconstructed NqrB–D complex was embedded in a 1-palmitoyl-2-oleoyl-sn-glycero-3-phosphocholine (POPC) phospholipid bilayer using CHARMM-GUI. A topology for POPC was obtained from the work of Kulig et al*.* [28] and used for all OPLS-AA/L simulations. Topologies for NqrB and NqrD were constructed using the ‘pdb2gmx’ routine in GROMACS. As in system ‘C’, the membrane-embedded protein complex was solvated with TIP3P water [29] and potassium chloride was introduced to an approximate 150 mM concentration. A 250 ps equilibration procedure at 310°K was performed following steepest descents minimization to machine precision. The integration timestep was increased from 0.5 to 1 fs after the first 50 ps, then to 2 fs during the production simulation. Protein coordinates were positionally restrained during the first 100 ps of equilibration.

### Micelle system preparation: simulations ‘M1–2’

These systems represent the initial investigation of the dynamics of the NqrB–D complex. Refinements introduced in the preparation of systems C and R1–3 later were absent from their design. The reconstructed NqrB–D complex was placed among pairs of POPC molecules using the GROMACS ‘solvate’ routine, then solvated again with TIP3P water. The initial positioning of POPC molecules in these systems, while not random, was effectively arbitrary. It resembled that of a bilayer but was unable to maintain such a configuration. POPC molecules were introduced in tail to tail pairs that were geometrically ordered with respect to one another. These molecules rapidly aggregated into micelles at the outset of the simulation. Two chloride ions were added to neutralize the amine terminals of NqrB and NqrD. Steepest descents minimization was carried out prior to production, but no equilibration step was performed.

### Simulation parameters

Table 2 summarizes key run parameters that differed between systems. All simulations were carried out under periodic boundary conditions in an orthorhombic simulation cell. Systems R1–3 and M1–2 used the OPLS-AA/L all-atom force field [30], while system C used the CHARMM force field [31] as implemented in CHARMM-GUI. The Particle–Particle Particle-Mesh algorithm with Analytical Derivative algorithm (P3M-AD) was used to describe long range electrostatic interactions under OPLS-AA/L, whereas the fast smooth Particle-Mesh Ewald (PME) algorithm was used for the CHARMM simulation. Both of these algorithms are discussed in the GROMACS User’s Manual [32]. The CHARMM simulation used a reciprocal grid of 96 × 96 × 120 cells with 4th order B-spline interpolation. The LINCS algorithm [33] was used to constrain bonds involving hydrogen atoms. All simulations were carried out at a temperature of 310°K and pressure of 1 bar. Temperature coupling was performed using velocity rescaling with a stochastic term [34]. Semi-isotropic pressure coupling was maintained for membrane-bound simulations, while isotropic coupling was used for simulations M1 and M2. The Berendsen barostat [35] was used to maintain pressure coupling during both equilibration and production steps because other options were found to be unusable for our system, hardware and software configuration.

**Table 2:** Run parameters for the six molecular dynamics simulations

System	Force	Duration	Integration	Van der Waals	Electrostatics		Neighbor search
	Field	(ns)	Timestep (fs)	Cutoff (nm)	Algorithm	Cutoff (nm)	Frequency (steps)
C	CHARMM	1000	2	1.200	PME	1.2	20
R1	OPLS-AA	150	2	1.321	P3M-AD	1.0	120
R2	OPLS-AA	150	2	1.321	P3M-AD	1.0	120
R3	OPLS-AA	150	2	1.321	P3M-AD	1.0	120
M1	OPLS-AA	50	1	1.225	P3M-AD	1.0	120
M2	OPLS-AA	50	1	1.217	P3M-AD	1.0	120

The micelle systems, not having been equilibrated, required a shorter integration time step. Van der Waals force cutoffs for the OPLS-AA/L systems were adjusted automatically on the basis of system characteristics

### Data analysis

Ramachandran plots and other validation data were obtained using the MolProbity server [36]. Data from six production simulations were collected. The total energies of each system were inspected to confirm stationarity. Visual Molecular Dynamics [37] was used to visualize trajectory data and compute backbone RMSD following alignment with the initial reconstructed coordinates. Descriptive statistics for each trajectory were then obtained using Octave [38].

Alignments and backbone RMSD values for the putative ubiquinone binding site at the NqrB–D interface were also computed. This site was defined on the basis of docking studies performed by Tuz et al. [16] using contacts of UQ-1 from the four lowest energy poses. UQ binding site residues on NqrB were defined as L181, T184, F185, V189, F193 and F211. On NqrD, the UQ binding site residues were defined to be F151, L155, L180, F189, L190 and F193.

Alignments and RMSD values for reconstructed regions were computed in a similar fashion for systems R1–3, but side-chain non-hydrogen atoms were included in the RMSD calculation. The RMSD density function for each reconstructed region in each trajectory was fitted to a Gaussian mixture model (GMM) in QtGrace v0.2.5a. Each GMM was a linear combination of no more than three Gaussian functions. GMMs were adjusted to maintain a correlation coefficient ≥ 0.98 while eliminating as many degrees of freedom as possible. For each region, Gaussian components belonging to different systems with centroids no more than 0.2 Å apart were iteratively clustered. The trajectory of the reconstructed region E164–G179 on NqrD was further analyzed using the gromos algorithm [39].

## Results

### System composition

A molecular dynamics simulation system was constructed with the objective of representing the behavior of NQR-inhibiting quinone analogs and NQR subunit amino acid side-chains at the NqrB–D interface. In order to accomplish this, it was necessary to include all residues in NqrB and NqrD that are in contact with residues from the other subunit. These residues would then have to be positionally restrained in a manner that approximates the influence of the surrounding protein scaffold on their mobility.

The mechanism of action of the Na^+^-NQR is not fully characterized. It is therefore difficult to deduce what manner of restraints would allow for a functional representation of the NqrB–D interface without introducing artificial rigidity. The restraining forces, therefore, had to be represented by including the ordered portions of subunits B and D. However, no attempt was made to introduce predicted disulfide linkages into the model in order to avoid assumptions that may introduce artificial constraints on protein motion.

Poorly ordered loop regions can also contribute to protein function but are difficult to visualize with X-ray crystallography. In particular, residues L167–P176 of NqrD are found in proximity to the NqrB–D interface and may influence the behavior of small molecules at the interface between these two subunits. Meanwhile, loops between the ordered elements of a subunit could serve to maintain appropriate mechanical coupling within the scaffold. Non-terminal loop regions were, therefore, interpolated and included in the model. Of note, the FMN cofactor covalently bound to Thr236 on one of the reconstructed regions of NqrB was excluded from the model because a residue topology for Threonine-FMN was not available.

It was uncertain whether the inclusion of a lipid bilayer would be required to maintain structural integrity at the NqrB–D interface or with what fidelity such a bilayer may need to be represented should one be required. Three system types were, therefore, investigated: one with a wide, explicitly defined nativistic bilayer around the subunits (system ‘C’), a second option with a compositionally simplified and spatially truncated bilayer representation (systems ‘R1–3’) and a third where phospholipids would be present to stabilize exposed hydrophobic regions without necessarily forming a bilayer (systems ‘M1–2’). These are further described in the respective system preparation sections.

The various subunits of Na^+^-NQR may influence the dynamics of any other subunits via long-range interactions. However, long-range interactions would propagate across the complex at timescales that are slow relative to dynamics of small molecules and side chain conformational changes at the NqrB–D interface. They were therefore excluded from the model.

### Reconstruction quality

**Fig. 1:**
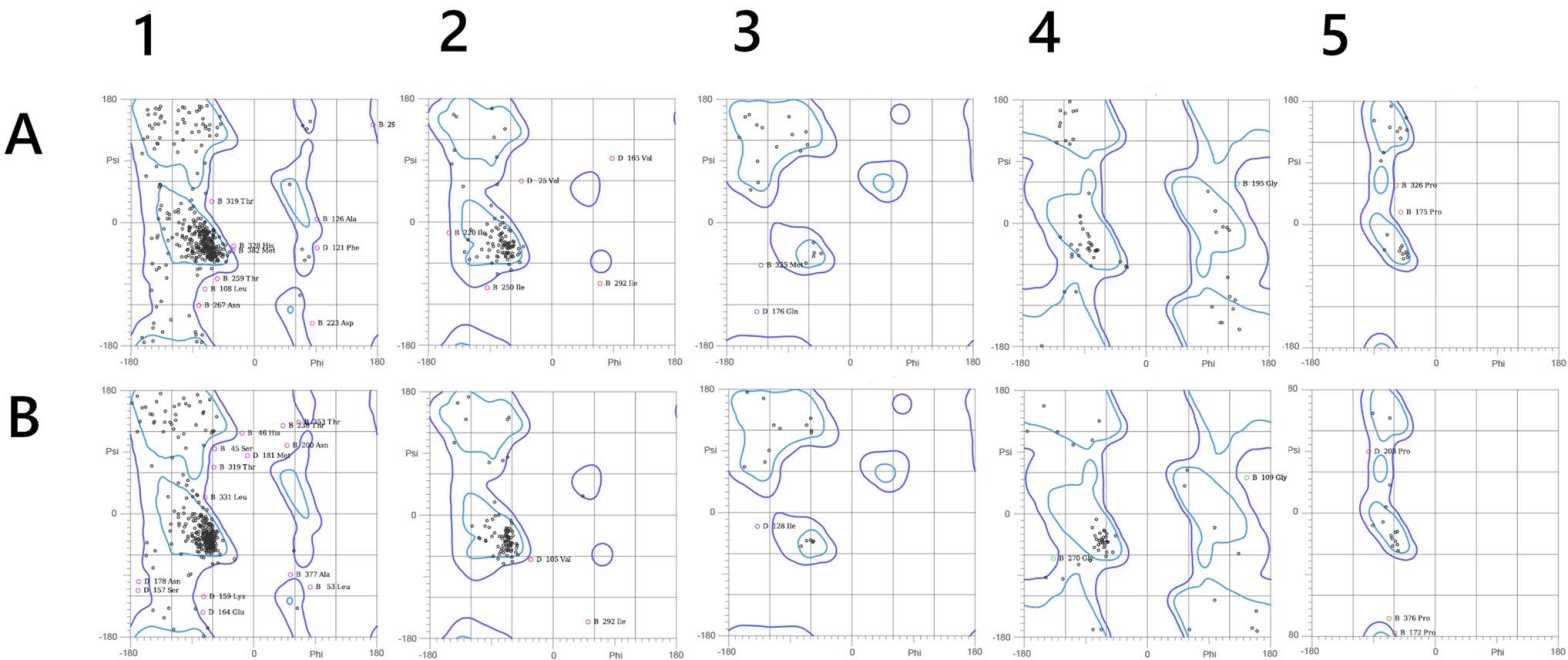
Ramanchandran plots of the reconstructed (**A**) and original (**B**) crystal structures. **1** General case, **2** isoleucine and valine, **3** pre-proline, **4** glycine, **5** *trans-*proline. There were no *cis-*proline residues in either structure

**Fig. 2:**
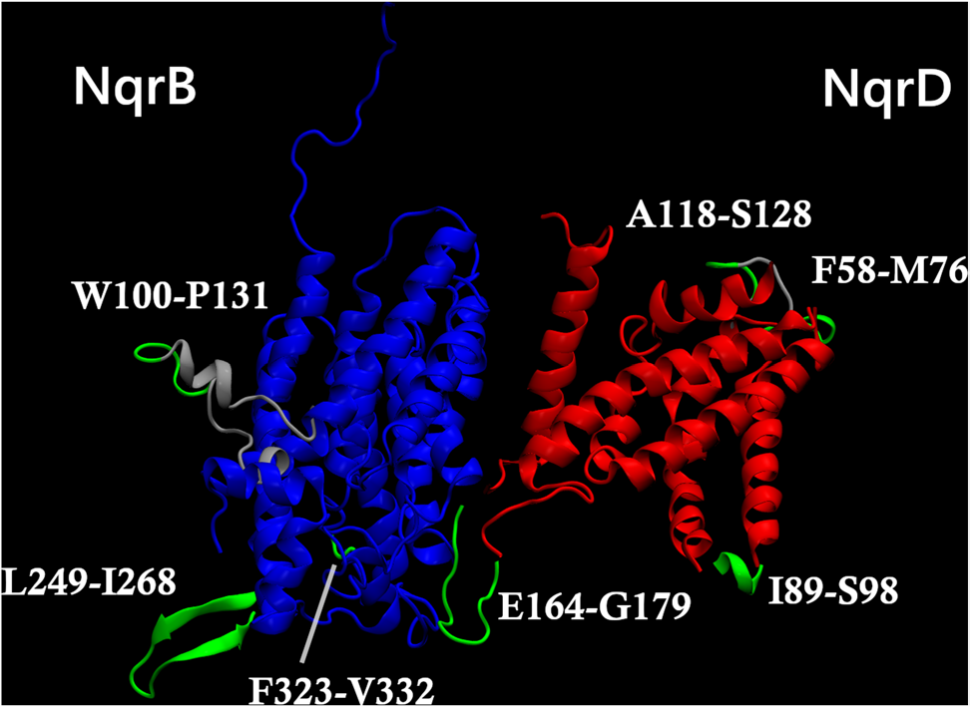
Reconstructed regions of the NqrB–D complex superimposed on a cartoon representation of the 2014 Steuber et al. [7] crystal structure. NqrB crystallographic coordinates are shown in blue, while NqrD is shown in red. Regions that were modified during algorithmic loop reconstruction are labelled. Existing residues that were considerably displaced during the reconstruction process are shown again in grey, while existing residues that underwent smaller displacement are omitted from the image for clarity. Residues that were absent from the original crystal structure but added during algorithmic loop generation are shown in green. The approximate positions shown are reflective of the NqrB–D complex after all reconstructive steps were completed

Ramachandran plots of NqrB and NqrD as reconstructed are compared to the crystal structure in Fig. 1. Figure 2 shows where essential features of the reconstructed structure differ from available crystallographic data. Despite the addition of new residues, the reconstruction had two fewer Ramachandran outliers (20) than the crystal structure (22). The crystal structure had 30.15 serious steric overlaps (> 0.4 Å) per 1000 atoms, while the reconstruction had 0.23. While there were no bond length aberrations, the reconstructive process introduced 78 new strained angles into the geometry. This is likely a consequence of the initial loop interpolation process.

### Descriptive statistical overview of trajectory kinematics

**Fig. 3:**
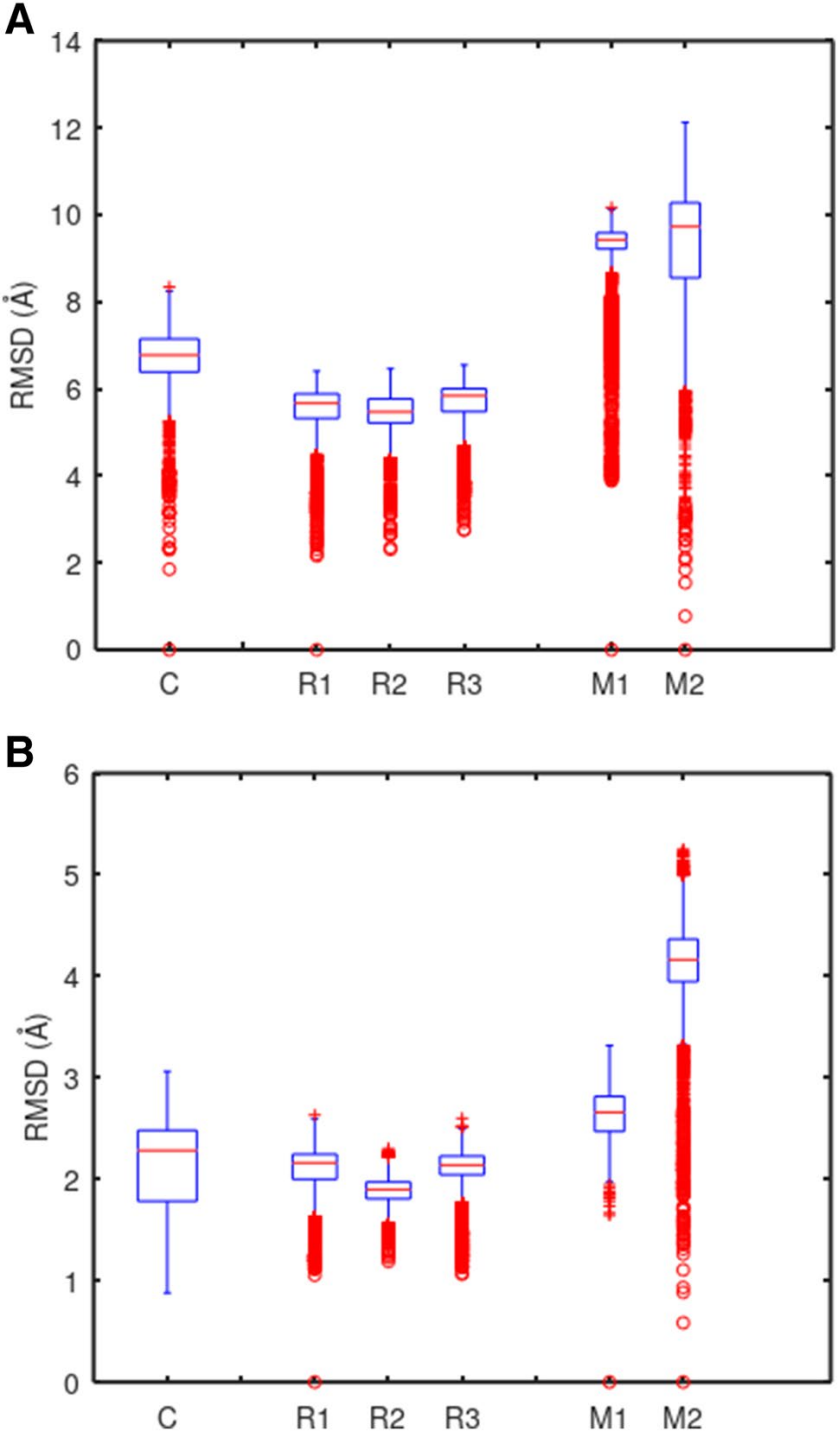
The root mean square displacement of all (panel **A**) and putative ubiquinone binding site (panel **B**) backbone atoms during each molecular dynamics simulation. Simulation ‘C’: CHARMM, membrane-bound, 2 fs timestep, 1 µs duration. Simulations ‘R1–3’: OPLS-AA/L, membrane-bound replicates, 2 fs timestep, 150 ns duration. Simulations ‘M1–2’: OPLS-AA/L, micelle systems, 1 fs timestep, 50 ns duration

Trajectory RMSD data for each of the six simulations is represented using individual box plots in Fig. 3, while Table 3 presents the RMSD medians alongside a mean and a 95% confidence interval on individual observations. The distributions of the data are left-tailed because each trajectory starts at an RMSD of zero, displacing from the initial coordinates until a steady state is achieved.

**Table 3:** Descriptive statistics for each of the molecular dynamics simulation trajectories

System	Protein RMSD			Binding site RMSD		
	Median (Å)	Mean (Å)	95% CI (Å)	Median (Å)	Mean (Å)	95% CI (Å)
C	6.78	6.75	1.17	2.28	2.15	0.80
R1	5.67	5.52	1.13	2.16	2.09	0.46
R2	5.40	5.47	1.04	1.90	1.87	0.31
R3	5.66	5.84	1.02	2.14	2.11	0.36
M1	9.12	9.43	1.88	2.66	2.64	0.44
M2	9.30	9.73	2.98	4.16	4.11	1.00

Simulation ‘C’: CHARMM, membrane-bound, 2 fs timestep, 1 µs duration. Simulations ‘R1–3’: OPLS-AA/L, membrane-bound replicates, 2 fs timestep, 150 ns duration. Simulations ‘M1–2’: OPLS-AA/L, micelle systems, 1 fs timestep, 50 ns duration

While the considerable variation between the two micelle system trajectories is noteworthy, direct comparison of intragroup differences between the OPLS-AA/L micelle and membrane runs on the basis of these descriptive statistics alone is challenging. The two micelle systems were built with different water–phospholipid ratios, while the OPLS-AA/L membrane runs are replicate runs of identical starting systems. With regard to intergroup differences between the three system types, it is important to keep in mind that the total simulation durations differ between the groups. Such comparisons are better enabled by direct examination of simulation time courses.

### Trajectory of the CHARMM system over one microsecond

**Fig. 4:**
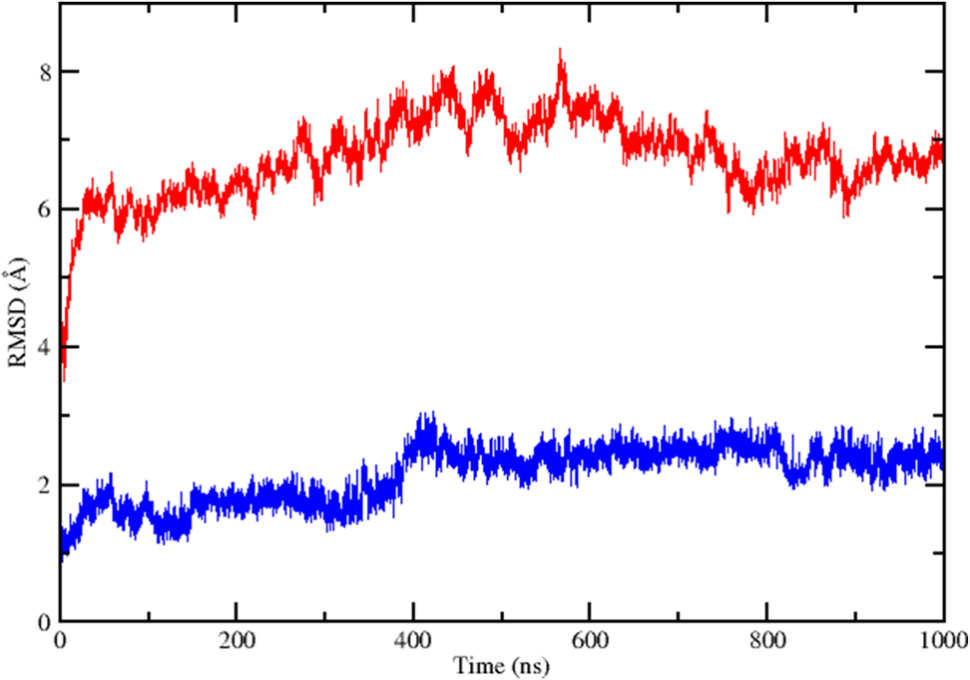
Embedded in a nativistic bilayer, the *V. cholerae* NqrB–D complex remained grossly stable for the duration of a 1 µs CHARMM molecular dynamics simulation at 310°K. However, a putative ubiquinone binding site located at the interface between the two subunits underwent a non-physiological transition after 350 ns

Table 4 summarizes salient events that occurred in the course of the microsecond CHARMM simulation, while Fig. 4 showed the protein and UQ binding site backbone RMSD from the initial conformation as a function of time. The complex of NqrB and NqrD was grossly stable over this duration, with modest linear drift of the UQ binding site backbone complete by approximately 35 ns.

**Table 4:** Summary of salient events observed during a 1 µs CHARMM molecular dynamics simulation at 310°K

Time (ns)	Event	UQ site	Total
		RMSD (Å)	RMSD (Å)
0	Start	1.0	2
35	UQ site linear backbone drift complete	1.5	6
260	NqrB V193 and NqrD F193 contact lost	1.8	7
350	UQ binding site deformation begins	1.9	6
440	UQ site deformation complete	2.5	8
1000	End	2.4	7

Corresponding RMSD values for ubiquinone binding site residue and whole protein backbone atoms are presented. The start coordinates were not identical to those of the input system due to displacement during the initial equilibration steps

**Fig. 5:**
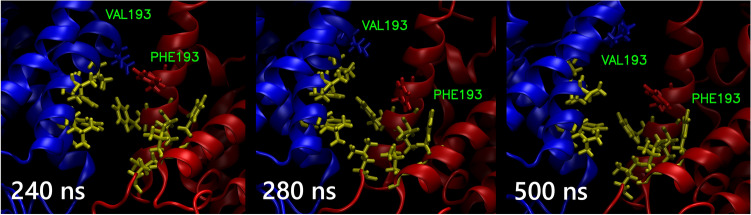
Visualization of the putative ubiquinone binding site at the interface between NqrB and NqrD. V193 of NqrB is shown in blue, while F193 of NqrD is shown in red. The remaining binding site residues are represented in mauve. Ghosted cartoon images of subunit B and subunit D are colored blue and red, respectively. The sequence of three images first shows loss of contact between NqrB V193 and NqrD F193 (240–280 ns). This was followed by further deformation, with nearby side chains on NqrB rotating away from the remainder of the site (280–500 ns)

Despite this overall stability, significant deformation at the UQ binding site occurred during the simulation. Figure 5 represents a visualization of this process. Contact between NqrB V193 and NqrD F193, present in the initial conformation, was lost after 260 ns. A wider binding site deformation process could be inferred from the aggregate RMSD of all binding site residues around 350 ns. The remainder of this process lasted approximately 90 ns. One coil of the NqrB α-helix housing the binding site residues of this subunit unwound as the site deformed. Given the spatial requirements of this change, it suggests non-physiological behaviour.

### The 150 ns trajectory of a membrane-bound NqrB–D complex is comparable in CHARMM and OPLS-AA/L

**Fig. 6:**
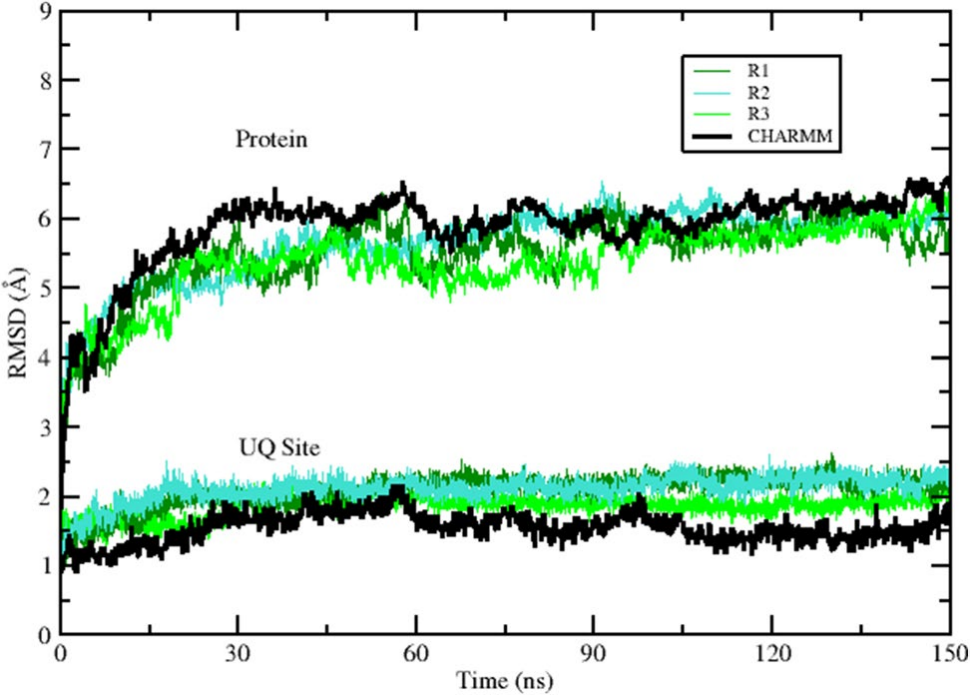
During the first 150 ns of simulation, three replicates (R1–R3) of an OPLS-AA/L system where the NqrB–D complex was embedded in a truncated POPC bilayer followed similar trajectories. Furthermore, their behavior is consistent with that of a larger CHARMM system where the complex was embedded in a nativistic bilayer

Three replicates of the same OPLS-AA/L membrane system were run 150 ns each to evaluate their congruence with the CHARMM trajectory. This comparison is shown in Fig. 6. The OPLS-AA/L system is stable between 30 and 150 ns, qualitatively representing a fair approximation to the more nativistic CHARMM system. While the CHARMM system had an 80% POPE and 20% POPG composition, the OPLS-AA/L systems had a 100% POPC membrane composition and were constructed in a smaller simulation cell.

### OPLS-AA/L micelle-forming systems produce less reliable trajectories

**Fig. 7:**
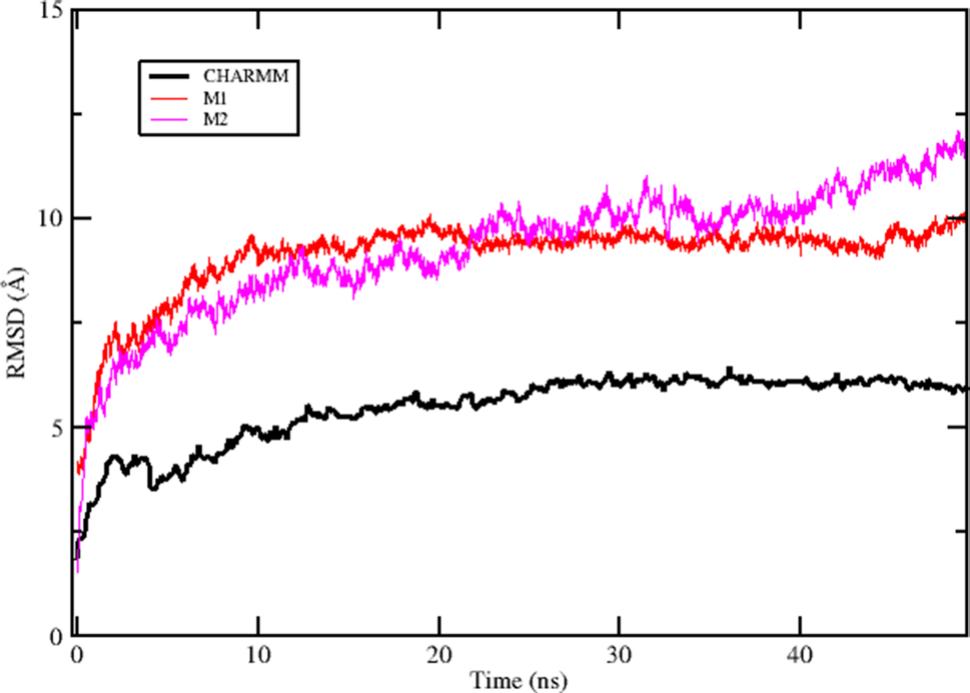
NqrB–D complex stability was poorly preserved in micelle systems at 310°K. Whole protein backbone RMSD is shown for the two OPLS-AA/L micelle systems (M1–M2), with that of the CHARMM system presented for comparison. It was unclear if M2 reached a steady state by the end of the simulation at 50 ns

Two OPLS-AA/L micelle systems were run for 50 ns each. Unlike the membrane-bound complex, they showed poor preservation of starting coordinates. Figure 7 compares the whole protein backbone RMSD of the OPLS-AA/L micelles system with that of the membrane bound CHARMM system during the first 50 ns of simulation.

UQ binding site coordinates were also disrupted by the non-micelle milieu. With no bilayer to provide a shared hydrophobic restraint for the two subunits, slippage between them could be observed in system M2. Packing density at the NqrB–D interface increased in both systems, with the binding site residues moving to make closer contact with one another.

### Reconstructed region dynamics

Due to the absence of relevant crystallographic data, three regions of NqrB and four regions of NqrD were reconstructed. The reconstructed regions of NqrB spanned residues W100–P131, L249–I268 and F323–V332. In NqrD, the regions F58–M76, I89–S98, A118–S128 and E164–G179 were reconstructed. The length of the reconstructed regions exceeded that of gaps in the crystal structure because PyFREAD modifies the coordinates of flanking residues to better accommodate the newly modeled component.

The behavior of reconstructed regions in systems R1–3 was examined using a unidimensional clustering procedure. The RMSD density function of each region in each system was fit to a GMM, and the resulting modes were clustered across systems. Table 5 summarizes those clusters that contained more than one member.

**Table 5:** Components extracted from the GMM analysis of reconstructed region RMSD density of states functions

Subunit	Region	*µ*	*σ*	AUC
		(Å)	(Å)	
NqrB	W100–P131	4.4	0.38	0.77
		3.3	0.62	0.38
	L249–I268	4.1	0.61	0.91
	F323–V332	1.6	0.37	0.63
NqrD	F58–M76	1.5	0.38	0.59
		2.0	0.36	0.60
	I89–S98	3.7	0.63	0.60
	A118–S128	2.9	0.45	0.86
	E164–G179	4.2	0.54	0.53
		4.6	0.31	0.31
		3.6	0.42	0.22

*µ*, *σ*—mode and standard deviation of the Gaussian distribution, respectively. AUC—area under the curve of a GMM component normalized by the total area under the curve of the GMM, averaged between systems. AUC values by region do not sum to unity because different component modes predominated in different systems. A component was treated as significant if it shared a mode within 0.2 Å with a component from a different replicate

W100–P131 of NqrB consisted of an N-terminal loop lying against a C-terminal helix. This substructure was flanked by helical elements. L249–I268 was a beta sheet, while F323–V332 did not have an ordered secondary structure. Two distinct states were identified for the W100–P131 region of NqrB, while the RMSD distributions of L249–I268 and F323–V332 were each described by a unimodal function. Although the ten amino acid F323–V332 loop was less mobile than the 29 amino acid L249–I268 beta sheet, the conformational state space of the former was more nuanced. Whereas the 4.1 Å mode of L249–I268 accounted for an average 91% of the cumulative RMSD density for this loop, the 1.6 Å mode of F323–V332 accounted for an average of only 63%.

In NqrD, the regions F58–M76, I89–S98, A118–S128 were each comprised of a loop flanked by helical elements. A significant portion of both flanking elements was included in F58–M76. An N-terminal helical element spanning one full turn was included in A118–S128. The I89–S98, in the meantime, assumed a cloverleaf shape and included only half a turn of helix at the N-terminal. Region E164–G179 of NqrD showed an entirely disordered secondary structure but exhibited nuanced behavior. Three distinct structural states were identified for the E164–G179 region. The GMM components describing these states were centered on RMSD values of 4.2 Å, 4.6 Å and 3.6 Å.

**Fig. 8:**
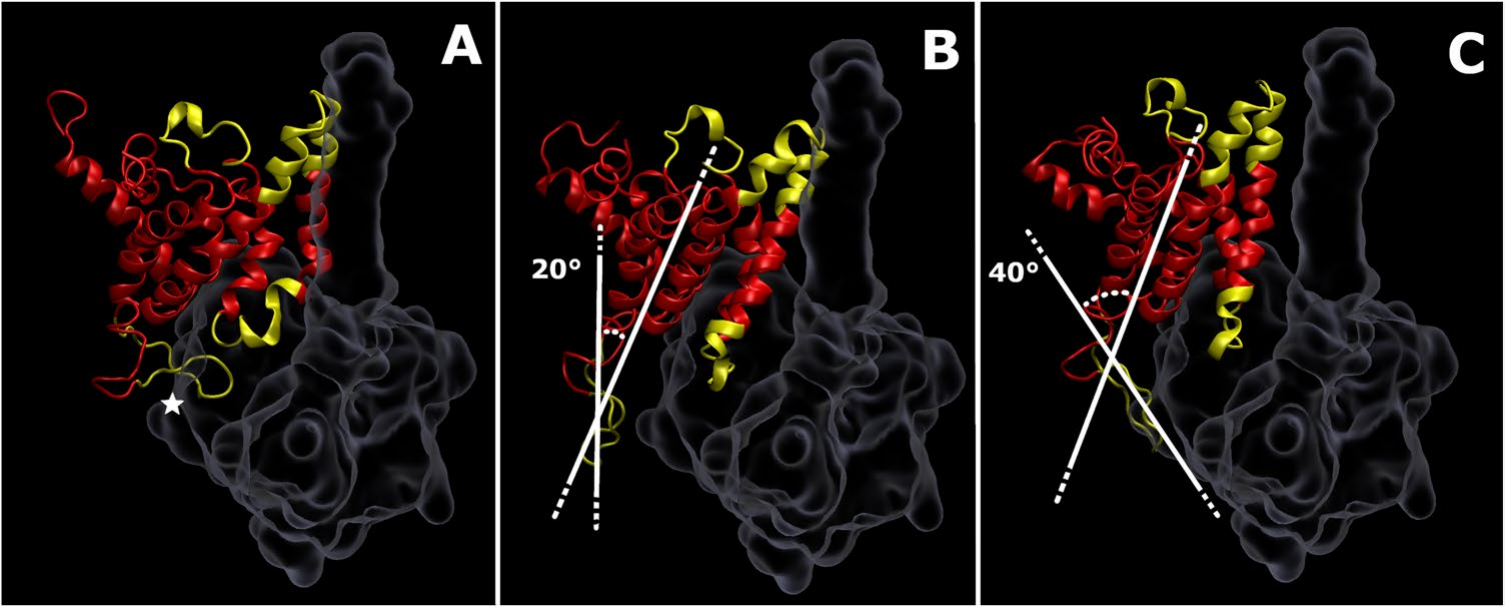
Structural clusters of the E164-G179 reconstructed region in NqrD from the combined trajectory of systems R1–3. NqrD is shown as a red cartoon, with yellow areas representing reconstructed regions. NqrC was not explicitly modeled, but the approximate surface of this subunit is shown as an outline. Panel **A** shows a nonphysical conformation, with the steric clash between NqrC and the loop marked by a five-pointed star. Panel **B** shows the ‘open’ conformation of the reconstructed region, while panel **C** shows the ‘closed’ conformation. The acute angle made by the major axis of the reconstructed region and the axes of the transmembrane helices is shown for both physical conformations

Given the relatively complex behavior of this reconstructed region, an alternative clustering analysis was performed using the gromos algorithm. This algorithm uniquely and explicitly assigned frames of the trajectory to a cluster, allowing for the extraction of structures that are representative of each. The three most populated clusters returned by this method qualitatively corresponded to those produced by the GMM, with central RMSD values of 4.4 Å, 4.5 Å and 3.6 Å. The former two clusters each accounted for 24% of frames from the pooled R1–3 trajectories, while the latter accounted for 17%. Figure 8 shows a representation of these three modes. The 4.5 Å mode was not physical, with the reconstructed loop overlapping with the location of NqrC in the complete NQR complex. The physically plausible modes differed from one another with regard to the angle of the reconstructed loop relative to the axes of the surrounding helices. The salient angle is measured in the plane by the intramembrane minor axis and transmembrane axis of the NQR complex.

The 4.4 Å mode represented an ‘open’ conformation of the loop, with minimal angulation between its major axis and that of the α-helices immediately surrounding it. This conformation exerts few, if any, steric effects on NqrC. In contrast, the 3.6 Å mode positioned the loop in a ‘closed’ conformation. This conformation is distinguished by an angle of 40° between the loop and surrounding helices. The resulting minor steric clash with NqrC would restrict the relative mobility of NqrC with respect to NqrD at the periplasmic surface of the NQR complex.

## Discussion

The present data demonstrates that an abbreviated molecular dynamics system containing only NqrB and NqrD embedded in a phospholipid bilayer surrounded by a 0.15 M KCl solution is adequate for the study of small molecule behavior at the NqrB–D interface. This was accurate on a timescale of hundreds of nanoseconds at 310°K. The primary evidence presented in support of this conclusion is the stability of the NqrB–D complex at this timescale in two different force fields, as well as the congruence between the CHARMM and OPLS-AA/L coordinate trajectories. This conclusion is strengthened by counter examples when system stability is not maintained. Namely, the complex did not retain a near-native configuration in simulations without a bilayer present and at timescales where helix-coil transitions that are sterically hindered by absent members of the complex become possible (in this specific case, times exceeding ~ 250 ns).

The validation of a smaller molecular system using a larger and more complex system will be important for future in silico modeling of drugs that inhibit Na^+^-NQR, as the exclusion of extraneous elements allows for improved computational efficiency without loss of accuracy. In the present study, production rates of 141 ns per day were achieved for the CHARMM system, whereas the spatially abbreviated and compositionally simplified membrane-bound OPLS-AA/L system allowed for rates of 278 ns per day.

This work also reported on three innovative techniques. The first was a method for the reconstruction of protein structures suitable for atomistic molecular dynamics simulation from incomplete crystallographic data. An in-house implementation of FREAD was first used to reconstruct protein loop backbones. The molecular modeling program Nanome was then used to rebuild missing sidechains and sculpt the protein structure in virtual reality. The repair of steric clashes and irrational structural elements introduced by algorithmic loop generation was informed by chemical intuition.

The behavior of the reconstructed regions was described using a unidimensional clustering method. While two-dimensional GMM clustering methods have been used previously to describe free energy landscapes [40], the use of a GMM to resolve an RMSD density of states function into components has not previously been described in the literature and represents another procedural innovation. Beyond describing the RMSD state space within a single replicate, the integral of each Gaussian component and the propensity of centroids to recur between replicates provides insight into the relative exhaustiveness of the state space search. Components that recur between replicates and account for a nontrivial portion of the state space can then be treated as significant.

Since it is simpler than other clustering methods [41], it may be possible to effectively use a more automated implementation of this unidimensional clustering procedure to screen the entirety of a protein for elements that exhibit well described, salient regional behaviors within a simulation. However, the procedure carries two intrinsic disadvantages. The first of these is an inability to resolve disparate clusters that have a high RMSD from one another, yet share a similar RMSD with respect to the frame of reference. In practice, where such components are significant, they would likely differ in their width. This would cause the model to use two overlapping Gaussians to represent the relevant region of the RMSD state density, resolving the two clusters. However, should the spread and centroid of these components be identical, they would be impossible to separate. The second disadvantage is an inability to extract a set of representative coordinates for each component directly from the model.

The unidimensional clustering procedure was used to scout the state space of small structural completions. Three recurring state clusters were identified for the E164-G179 region of NqrD. Of all the reconstructed regions, this one exhibited the most complex reproducible behaviors. To investigate its behavior further and to validate the unidimensional clustering procedure, a conventional clustering analysis was performed on this region. While this analysis showed a qualitative correspondence to the GMM, the top three clusters accounted for less of the state space than each of the corresponding GMM components. Thus, while the unidimensional GMM did provide insight into the density of states within the hyperdimensional state space of the coordinate system, the simple model could not adequately resolve the fine structure of this space. Further comparison of unidimensional clustering results with results from conventional clustering procedures for systems of different size and composition will ultimately be required to fully establish the utility of this procedure.

Excluding the artefactual state where the loop of E164-G179 folds to lie against the periplasmic face of NqrD, this loop was observed in two major conformations. One of these—the ‘closed’ conformation—follows the notional contours of NqrC. The other—an ‘open’ conformation—has the loop resting just beyond the contours of the unmodeled NqrC subunit. Transitioning from the open to the closed conformation involves a 20° flexion of the E164-G179 loop that brings it into contact with NqrC. Taken together, this suggests that the E164-G179 region of NqrD acts as a sort of latch that anchors it against NqrC.

While none of these conformations bring the E164–G179 region into a position where it can directly interact with the ubiquinone binding site at the NqrB–D interface, it is conceivable that the behavior of this region may change in the presence of ubiquinone or inhibitors of the NQR complex. Pulling the C-terminal polar residues Gln176 or Asn178 closer to the ubiquinone binding site, for example, would necessitate a conformational change at the NqrC–D interface.

The third innovation created in the present investigation was the use of a GPU halo exchange feature for molecular dynamics simulation. GPU halo exchange is considered an experimental feature in the 2020.3 version of GROMACS [21, 32, 42]. It was used successfully in simulations C and R1–R3 in order to distribute all coordinate update tasks between two NVIDIA GPUs for the duration of a single run. Simulations M1–2 were performed before the NV Link bridge was installed, so GPU halo exchange was not used for them.

These procedural steps produced well-behaved systems that yielded chemically reasonable results. However, the potential limitations of these procedures will necessitate further investigation. For example, our aggressive use of GPU acceleration limited available thermostat and barostat choices. The use of the Berendsen barostat should ideally be avoided in production simulations. This particular limitation is likely to be overcome by the anticipated introduction of the stochastic cell rescaling [43] barostat in the 2021 release of GROMACS, which should be similar in implementation to the Berendsen barostat.

Exploring alternative loop completions, documenting any artefacts produced by GPU halo exchange and measuring the inter-operator reliability of manual protein structure reconstruction in VR would allow for a better understanding of the strengths and weaknesses of these new methods.

A number of other limitations are considerations in the current work. The most serious of these is a lack of experimental data to corroborate simulation results. While convergent evidence from two in silico approaches strengthens our conclusions, the suitability of any model is ultimately determined by its ability to describe empirical observations. Although the membrane-bound systems presented here are well-behaved, no direct comparison with experimental in vitro data is possible at this time.

Another limitation may emanate from the heterogeneity of the collected data. Using identical integration time steps, run times and system preparation methods would doubtless enable more nuanced comparisons of intergroup data. Given the data were collected at various stages of hardware deployment and software toolchain refinement, such heterogeneity was inevitable. Care was taken, therefore, to present appropriate comparisons between the data. Statistical comparisons were avoided for the assumptions that did not fit the underlying paradigm.

Despite its general stability, our representation of the NqrB–D complex is limited by the absence of disulfide linkages. A special residue is also incorrectly represented (Thr236 on NqrB is missing a bound FMN cofactor). While no obvious problems arose as a result of these omissions, the structural stability of the model can likely be improved by incorporating these missing features. However, such a refinement is likely to have only a minimal impact on computational efficiency.

Other structural refinements would negatively impact the computational performance of the model despite improving its fidelity. For example, the incorporation of NqrA into the protein complex can be expected to introduce steric effects that prevent conformational disfigurement at the cytoplasmic aspect of the NqrB–D interface 260 ns into the simulation. Moreover, the interface between NqrB and NqrA is itself of considerable interest. Masuya et al. [44] used photoaffinity labeling studies to document inhibitor binding near a ubiquinone binding site [45] at this interface, proposing that the residues identified by Tuz et al. [16] influence ubiquinone binding via indirect effects that lead to long-range structural changes.

In spite of its limitations, this model along with the methods used to construct and validate it have three immediate applications. Firstly, the reconstructive methodology used to produce the NqrB–D interface model can be applied to investigation of the NqrA–B interface. Indeed, a complete model of the *V. cholerae* NQR complex can be reconstructed and represented with iterative loop reconstruction and manual sculpting. Simulating a complex of this size is computationally expensive but may be possible using coarse-graining methods [46].

Secondly, in silico mutagenesis targeting key residues of NqrB and NqrD can be used to investigate structure–activity relationships in the NQR complex. The relationship between the higher order structure of the NqrB–D interface and experimentally measured NQR catalytic activity can be investigated for cases where the assumption of secondary structure homology between the wild type and mutant variants can be sustained. An assumption of secondary structure homology must first be validated outside of this model because the resulting system is not expected to retain stability at timescales where helix-coil transitions take place. The target residues in a system based on the ‘R’ series would then be modified to reflect the mutation of interest. The resulting system would then be equilibrated and run in the fashion described here, enabling a geometric comparison between wild type and mutant complex trajectories.

Finally, this model can be used to probe the behavior of NQR complex inhibitors such as korormicin at the NqrB–D interface. Preliminary work in this direction by our group suggests that a major challenge in using this model for the study of protein–ligand interactions lies in the prevention of nonspecific binding to hydrophobic surfaces that would normally be inaccessible due to the presence of other NQR subunits. While the use of micelle rather than membrane-bound systems is a technically simple strategy for protecting exposed hydrophobic surfaces, we have shown that the NqrB–D does not retain stability reliably in micelle systems. Several alternative system preparation strategies are being investigated further by our group.

## Data Availability

Data is available upon reasonable request to the author.

## Code availability

The modified PyFREAD code is available upon reasonable request to the author.
